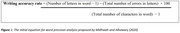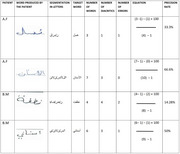# A novel approach in quantifying writing errors in Arabic‐speaking patients with Alzheimer's disease

**DOI:** 10.1002/alz70857_100268

**Published:** 2025-12-25

**Authors:** Mohamed Taiebine, Mustapha El Alaoui Faris, Maria Benabdeljlil, Saadia Aidi, Mounia Rahmani, Khadija Al Zemmouri

**Affiliations:** ^1^ Euro‐Mediterranean University of Fez (UEMF), Fez, Morocco; ^2^ Alzheimer's center, Rabat, Morocco; ^3^ Faculty of Medicine and Pharmacy, University Mohammed V, Rabat, Morocco; ^4^ Department of Neurology and Neuropsychology, Specialty Hospital, Rabat, Morocco

## Abstract

**Background:**

To our knowledge, few studies have been conducted in Arabic regarding cognitive‐linguistic characterization of acquired written language disorders (agraphia) in neurodegenerative diseases, while the majority of them has been carried out in English and Indo‐European languages. In this study, we characterized agraphia in Alzheimer's disease (AD).

**Method:**

The study sample consisted of 14 patients (4 with mild AD and 10 with moderate AD). They underwent the Moroccan version of the Mini‐Linguistic State Examination (MLSE) which is a screening battery for language disorders in neurodegenerative diseases (Taiebine et al.,2021, 2024). We used an equation to calculate the degree and type of accuracy in the graphemic realization of consonants and diacritics based on the work of Midhwah and Alhawary, (2020) (Figure 1).

**Result:**

According to our neurocognitive model of agraphia in Arabic (Taiebine et al., 2024), graphemic and post‐graphemic buffer errors are produced by patients with moderate AD. The application of the equation for analyzing writing accuracy during the MLSE writing subtest showed different patterns in inter‐ and intra‐individual performances. By averaging the accuracy rate for each patient, we identified 3 groups: those with a high accuracy rate (more than 65%); those with an average accuracy rate (between 40% and 65%) and those with a low accuracy rate (less than 40%). (Table 1)

**Conclusion:**

This study provides a new perspective in the neurolinguistic characterization of agraphia in AD. The typical pattern of graphemic disorders found in our patients is consistent with the impairment of concomitant triple peripheral writing buffers: graphemic, graphomotor, and allographic.